# Association between medication adherence to chronic diseases and shift-work schedules in the Korean working population

**DOI:** 10.1038/s41598-022-26618-9

**Published:** 2022-12-30

**Authors:** Seung-Yeon Lee, Seunghyun Lee, Wanhyung Lee

**Affiliations:** 1 Department of Family Medicine, International Healthcare Center, Seoul National University Bundang Hospital, Bundang-Gu, Seongnam-Si, Gyeonggi-Do Republic of Korea; 2 Department of Occupational and Environmental Medicine, College of Medicine, Gachon University, 21, Namdong-Daero 774, Namdong-Gu, Incheon, 21565 Republic of Korea; 3 Department of Occupational and Environmental Medicine, Gil Medical Center, Gachon University College of Medicine, 21, Namdong-Daero 774, Namdong-Gu, Incheon, 21565 Republic of Korea

**Keywords:** Occupational health, Patient education

## Abstract

Shift-working schedules are closely linked to chronic diseases, and only a few studies have investigated the association between working schedules and medication adherence in chronic diseases targeting workers. This study aimed to investigate whether shift-work schedules are associated with medication adherence in a working population with chronic conditions. The study participants (n = 11,460 person-years) were identified from the Korea Health Panel Study conducted from 2008 to 2018. Medication adherence was classified as good (2+) or poor (0 or 1) based on the 4-item Morisky Medication Adherence Scale. Work schedules were categorized as shifts and fixed daytime work. Its association with medication adherence was investigated using a generalized estimating equation model, generating odds ratios (OR) with 95% confidence intervals (CI). According to work schedules, shift workers were more likely than fixed-day workers to have poor medication adherence (adjusted OR = 1.16, 95% CI: 1.02–1.33). Regarding occupational classification, manual workers had a significantly higher risk of poor medication adherence than those in other occupational categories (adjusted OR = 1.27, 95% CI: 1.13–1.43). Among shift workers, the major reason for poor medication adherence was forgetting to take medication (SPR = 1.23, 95% CI: 1.07–1.38). Workers with irregular shift times are more vulnerable to poor medication adherence. Future studies are required to understand the mechanisms underlying this association and develop strategies to enhance medication adherence in the working population.

## Introduction

Chronic diseases are the leading cause of death worldwide, accounting for 71% of all deaths annually^1^. Medications are essential part of managing chronic diseases, and patients usually require long-term maintenance medications. However, a 2003 report on medication adherence by the World Health Organization (WHO) estimated that approximately half of the patients with chronic diseases do not take their medications as prescribed^2^. Similarly, a recent meta-analysis of eight studies involving 8,949 participants with two or more chronic conditions found that approximately 43% were nonadherent to their prescribed medications^3^. In this regard, improving medication adherence—following a medication treatment plan developed by an individual's healthcare provider and agreed upon by the patient by shared decisions, filling prescriptions, and taking medications as indicated—has long been a huge challenge for chronic disease management. Poor medication adherence is associated with disease progression, increased morbidity and mortality, and the increased use of healthcare services^4^. In contrast, good medication adherence is associated with improved clinical outcomes, such as reduced mortality and unnecessary healthcare expenditure^5^. Thus, ensuring that patients take their medications as prescribed is crucial for providing quality patient care, preventing further complications and disabilities, and reducing costs to healthcare systems and individuals.

Shift work is essential to meet the 24-h/7-day a week consumption of our society^6^. In 1990, the International Labor Organization defines shift work as "a method of organizing working time in which workers succeed one another at the workplace so that the establishment can operate longer than the normal working hours of an individual"^7^. Shift schedules can be structured in a variety of ways, including fixed (i.e., working the same shift every day) and rotating shifts (i.e., working different shifts on a cycling basis)^8^. In Korea, the number of shift workers is growing steadily, and a recent survey reported that approximately 10% of workers work in shifts^9^. As shift work becomes increasingly common, numerous studies have been conducted to investigate the effects of shift work on health. Circadian rhythm is a 24-h internal body clock that keeps our bodies in a regular routine in response to light and darkness. However, shift workers, who work outside normal daylight hours, confuse internal body clocks and lead to changes in numerous physiological processes^10^. Accumulating evidence suggests that circadian disruption in shift work is associated with an increased risk of developing various chronic diseases, mental disorders, and work-related accidents^11^. The successful management of such illnesses mainly depends on the medication and proper use as prescribed. Thus, a better understanding of the medication adherence status of shift workers and the factors that influence adherence will be of great significance for developing preventive strategies for the health of shift workers.

Medication adherence is typically measured either directly (e.g., observing pill consumption, measuring metabolites, detecting biomarkers, etc.) or indirectly (e.g., self-reporting, counting pills, using claim databases, electronic monitoring, etc)^12^. Each measure has advantages and disadvantages, and there is no gold standard. However, patient self-reporting is the most commonly used method in the clinical setting^13^. The Morisky Medication Adherence Scale (MMAS-4) is among the most widely used self-report measures for assessing medication adherence and its associated barriers. This has been validated in numerous studies^14^.

So far, there have only been a few studies on medication adherence targeting the working population^15^. Moreover, research on the association between shift work and medication adherence is limited. Therefore, this study aimed to investigate whether shift work was associated with medication adherence. In addition, we explored the reasons underlying poor adherence in this population using the 4-item MMAS.

## Materials and methods

### Data and study participants

**Figure 1:**
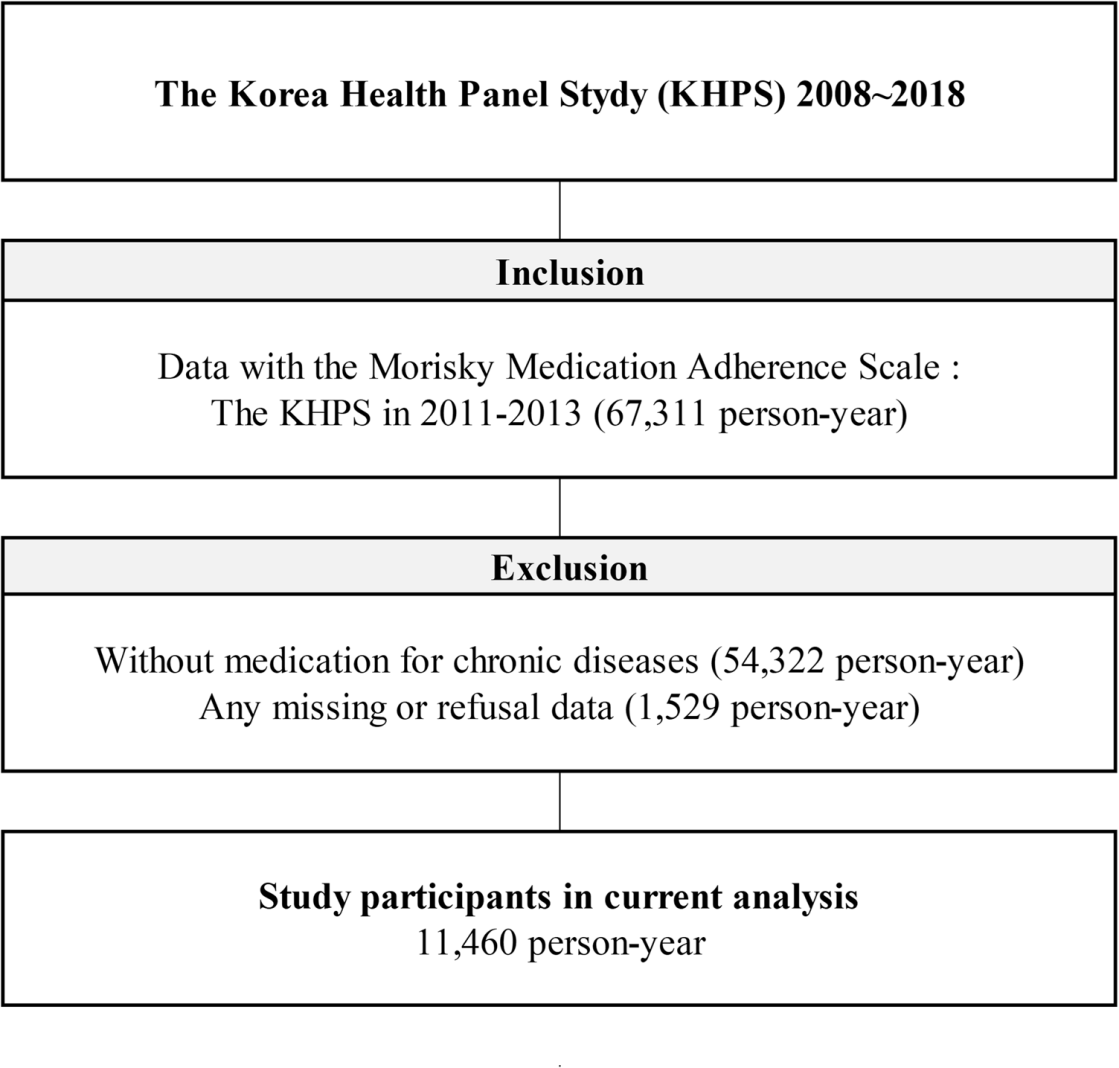
Schematic diagram depicting study population.

The Korea Health Panel Study (KHPS) is a survey of building panel data that can comprehensively analyze factors affecting medical use and expenditure, as well as information on medical use behavior and expenditure size^16^. All data are available upon request from http://www.khp.re.kr (accessed on April 13, 2022). We used the data from 2008 to 2018 (version 1.7.1). We selected 11,460 person-years from 67,311 person-years with information about medication adherence during the follow-up period. Figure 1 shows a detailed schematic representation of the study population. All data from the KHPS were collected after obtaining written informed consent from all the participants. The data in this study were anonymized before releasing the authors from the Korea Institute for Health and Social Affairs and the National Health Insurance Service. This study was approved by the institutional review board of the Gachon University Gil Medical Center (IRB number: GFIRB2022-045).

### Medication adherence

Medication adherence was assessed using four items from the MMAS-4^14,17^: “Do you ever forget to take your medicine?”; “Do you ever have problems remembering to take your medication?”; “When you feel better, do you sometimes stop taking your medicine?”; and “Sometimes, if you feel worse when you take your medicine, do you stop taking it?” The MMAS-4 uses a scoring scheme of “Yes” = 0 and “No” = 1. Therefore, the items were summed up to obtain scores ranging from 0 to 4. A score of 0 or 1 was considered poor adherence to medication^18^. In the current study, chronic diseases were defined as patients who needed medication for hypertension, diabetes, or dyslipidemia according to the Korean Standard Classification of Diseases.

### Occupational characteristics

The current study used occupational characteristics such as occupational classification and working schedule. The present analysis used a modified occupational classification with four categories from the ten major groups of the International Standard Classification of Occupations from the International Labor Organization, based on a previous study: office, service and sales, agriculture, forestry, fishing, and manual workers^19^. (1) Office workers included major group 1: legislators, senior officials, and managers; major group 2: professionals; major group 3: technicians and associate professionals; and major group 4: clerical support workers. (2) Service and sales workers include major group 5 (service and sales workers). (3) Agriculture, forestry, and fishing workers included in major group 6: skilled agricultural, forestry, and fishery workers. Finally, (4) manual workers consisted of major group 7: craft and related trade workers; major group 8: plant and machine operators and assemblers; and major group 9: elementary occupations. The Republic of Korea has assigned a minimum of 18 months of mandatory military duty to all male citizens (approximately aged 20–24 years). Thus, we used only nine major groups, excluding major group 0, because of the heterogeneity of age and sex for comparison with other countries. The working schedule was separated into two groups: day fixed and shift. The shift-work schedule included the entire working schedule, except for the fixed day.

### Covariates

The socioeconomic variables included age, sex, educational status, and household income level. Education level was classified as middle school, high school, college, or higher. Household income was divided into quintiles.

### Statistical analysis

We analyzed the prevalence of medication adherence according to baseline characteristics, occupational classification, and work type using the chi-squared test. In addition, we used questionnaires on medication for chronic diseases and related variables as repeated measurement variables from the KHPS during the follow-up period. Thus, we used the generalized estimating equation (GEE) to estimate the association between medication adherence to chronic diseases and shift-work schedules^20^.

The age-standardized prevalence ratio (SPR) and 95% confidence interval (CI) were estimated using all study participants in the current analysis as the reference population. To calculate SPR, age-specific poor adherence to medication for chronic disease prevalence was calculated for each 5-year age group among shift workers. These values were then multiplied by age-specific poor adherence to medication for chronic disease prevalence rates and the number of person-years in each age group of the study participants, which made the age-specific expected prevalence case among shift workers. The summation of all age-specific expected cases indicated the total prevalence. The ratio of the observed to the expected number of cases was the SPR. For sensitivity analysis, the SPR and 95% CI for poor adherence to medication for chronic diseases were calculated according to each item of the MMAS-4. The SPR and 95% CI for poor adherence to medication for chronic diseases were calculated for each item of the MMAS-4. All analyses were performed using Statistical Analysis System (SAS), version 9.4 (SAS Institute, Cary, NC, USA). For all statistical calculations, a two-tailed *P*-value of < 0.05 was considered statistically significant.

## Results

The general characteristics of the study participants according to their medication adherence are shown in Table 1. In a total of 11,460 person-years of observation, 65.8% (7,542 person-years) had good medication adherence and 34.2% (3,918 person-years) had poor medication adherence. Poor medication adherence was more common in women (35.1%), individuals aged 40 years or younger (42.1%), and those with a higher educational level than college (36.8%). Regarding work schedule, shift workers (36.5%) had a higher rate of poor medication adherence than fixed daytime workers. No significant differences in medication adherence were evident for household income or occupational classification.

**Table 1:** General characteristics of the study participants with chronic diseases according to medication adherence.

	Total, person-year (% of column)	Medication adherence, person-year (% of row)		*P*-value
		Good	Poor	
No. of participants	11,460 (100.0)	7,542 (65.8)	3,918 (34.2)	
**Sex**				0.0086
Male	4,508 (39.3)	3,032 (67.3)	1,476 (32.7)	
Female	6,952 (60.7)	4,510 (64.9)	2,442 (35.1)	
**Age (years)**				< 0.0001
≤ 40	986 (8.6)	571 (57.9)	415 (42.1)	
41–60	5,471 (47.7)	3,576 (65.4)	1,895 (34.6)	
> 60	5,003 (43.7)	3,395 (67.9)	1,608 (32.1)	
**Educational status**				0.0023
Middle school	6,143 (53.6)	4,106 (66.8)	2,037 (33.2)	
High School	3,189 (27.8)	2,092 (65.6)	1,097 (34.4)	
College or higher	2,128 (18.6)	1,344 (63.2)	784 (36.8)	
**Household income level**				0.9438
1st quintile	1,717 (15.0)	1,137 (66.2)	580 (33.8)	
2nd quintile	2,711 (23.7)	1,750 (64.6)	961 (35.4)	
3rd quintile	2,572 (22.4)	1,726 (67.1)	846 (32.9)	
4th quintile	2,293 (20.0)	1,512 (65.9)	781 (34.1)	
5th quintile	2,167 (18.9)	1,417 (65.4)	750 (34.6)	
**Occupational classification**				0.3225
Office	2,158 (18.8)	1,386 (64.2)	772 (35.8)	
Service and sales	1,786 (15.6)	1,157 (64.8)	629 (35.2)	
Agriculture, forestry, and fishing	2,072 (18.1)	1,444 (69.7)	628 (30.3)	
Manual	5,444 (47.5)	3,555 (65.3)	1,889 (34.7)	
Working schedule				0.0323
Daytime-fixed	10,338 (90.2)	6,829 (66.1)	3,509 (33.9)	
Shift	1,122 (9.8)	713 (63.5)	409 (36.5)	

In GEE models, shift workers were more likely than daytime-fixed workers to have poor medication adherence (adjusted OR = 1.16, 95% CI: 1.02–1.33) (Table 2). Regarding occupational classification, manual workers were at a significantly higher risk of poor medication adherence than those in other occupational categories (adjusted OR = 1.27, 95% CI: 1.13–1.43) (Supplementary Table 1).

**Table 2:** Results of a generalized estimating equation analyzing the risk of poor medication adherence.

	Model 1	Model 2	Model 3	Model 4
**Working schedule**				
Daytime-fixed	Reference	Reference	Reference	Reference
Shift	1.18 (1.03–1.34)	1.18 (1.03–1.34)	1.18 (1.03–1.34)	1.16 (1.02–1.33**)**

Model 1: adjusted age in observation year and sex.

Model 2: adjusted age in observation year, sex, and education.

Model 3: adjusted age in observation year, sex, education, and household income level.

Model 4: adjusted age in observation year, sex, education, household income level, and occupational classification.

Table 3 shows the SPR and 95% CI of the reasons for poor medication adherence based on MMAS-4. Among the four items of the MMAS-4, forgetting to take medication was significantly associated with poor medication adherence among shift workers (SPR 1.23, 95% CI: 1.07–1.38; P = 0.0047). Irregular daily routines and sleep habits in shift workers can be related to decreased alertness and forgetfulness, which may in turn affect poor medication adherence^15,21^.

**Table 3:** Age-standardized prevalence ratio (SPR) and 95% confidence interval (CI) of the reason for poor medication adherence according to each four items of the Morisky Medication Adherence Scale (MMAS-4) among shift workers.

	SPR (95% CI)	*P*-value
Do you ever forget to take your medicine?	**1.23 (1.07–1.38)**	0.0047
Do you ever have problems remembering to take your medication?	1.17 (0.88–1.47)	0.2537
When you feel better, do you sometimes stop taking your medicine?	0.90 (0.78–1.02)	0.1003
Sometimes, if you feel worse when you take your medicine, do you stop taking it?	1.05 (0.81–1.20)	0.9607

*Bold indicates statistical significance.

## Discussion

Our study found that shift work was associated with an increased risk of poor adherence to medication. This finding appears to be consistent with that of Sonoda et al.^15^, who reported two-fold higher odds of poor medication adherence in shift workers compared with non-shift workers; however, subjects were limited to male workers and the result was not statistically significant. In the present study, we included both genders and performed additional gender-stratified analyses to obtain information on the different conditions that women and men face as well as the different effects of work schedules on medication adherence.

The mechanisms underlying the association of shift work with medication adherence can be postulated based on some previous studies. Shifting work is a major cause of circadian stress. One of the most prominent and well-known examples of circadian stress in shift workers is the displacement of the sleep-wake cycle^22^. Poor sleep quality and quantity are associated with impaired alertness and decreased cognitive performance^21^, which may result in poor medication adherence. The gastrointestinal system also works via circadian regulation and functions poorly when circadian rhythms are disrupted by sleep displacement and irregular mealtimes^23^. Indeed, many previous studies have documented that gastrointestinal symptoms and diseases are more common in shift workers than that in normal daytime workers^24^. This may raise concerns regarding drug side effects, which are one of the factors influencing medication adherence^25^. In addition, given that hundreds of medications have mealtime-related dosing instructions, irregular meal habits among shift workers may be linked to medication adherence. A recent systematic review found that shift workers easily skip meals because of irregular daily routines and tiredness^24^. Sonoda et al. reported that irregular mealtimes and skipping meals are significantly associated with a higher risk of forgetfulness when taking medication during the working day^15^. Moreover, shift workers have difficulties combining their work and social lives^26^. They must sleep when others are awake, and they may find it difficult to visit hospitals regularly for disease management. This may have a negative effect on medication adherence.

In our study, manual workers had an increased risk of poor medication adherence compared with those in other occupational categories. Previous studies have shown that physical labor is associated with an increased risk of noncompliance with medication due to the relief of symptoms^27^. Our results on occupational health management insights help research and policies on making pill-taking more efficacious, providing health education, and improving verbal and nonverbal communication for the working population.

Our data showed that female workers were less likely to adhere to their medications. This could be related to the traditional sex role, where females are expected to serve as primary caregivers in the household, making them less likely to take care of their own health^28^. In addition, females are more likely to experience side effects from their medications^29^. Given that side effects are a common reason for discontinuing medications, experiencing more frequent side effects or fear of perceived side effects may lead to poorer medication adherence. Another possible reason for finding sex differences in adherence may be that women tend to experience more clinical signs and symptoms. Indeed, some studies have found that the female sex is associated with numerous and more severe somatic symptoms^30^. This can contribute to a loss of belief in the benefits of the medication^31^ and, as a result, poor adherence.

In Korea, males tend to dominate shift work and manual labor^32,33^, both of which were found to be associated with poorer adherence to medications in our study. With this in consideration, we conducted gender-stratified analyses to examine the interactions between gender and each variable as well as the association between poor medication adherence and work schedules (Supplementary Table 2, 3). There was no statistically significant difference in medication adherence between groups of occupational categories among both men and women. In terms of working schedule, it was observed that shift work was associated with a greater risk of poor medication adherence only in men. However, evidence of sex-based differences in medication adherence remains inconclusive. Manteuffel et al. and Granger et al. reported higher medication adherence in males than that in females, which is consistent with our results^34,35^. In contrast, Jahanpour et al. found that males have poorer medication adherence than that in females^36^. However, this study was conducted with a small sample size (n = 125), and only older patients (≥ 60 years) were included. Minaiyan et al. reported no significant association between sex and medication adherence^37^. The discrepancy among studies could be related to different cultures and societies because each study was conducted in a different country. Another possible reason for the discrepancy in the results may be the inconsistencies in the measurements (i.e., medication possession ratio, pill counts, or MMAS) of medication adherence. Thus, the findings on sex differences continue to be a source of speculation.

Previous studies have reported an association between socioeconomic status and medication adherence, but with inconsistent results^38^. Although our results did not show a significant association between socioeconomic status and poor medication adherence, further studies with more accurate and detailed information about vulnerable workers are required.

To the best of our knowledge, this is the first study in Korea to investigate the association between shift-work schedules and medication adherence in a working population with chronic illnesses. Additionally, our study is unique in that no previous study has investigated the differences in poor medication adherence across occupational categories. Moreover, the study population was derived from a nationwide representative panel survey, which allowed our findings to be generalized to a wider population in South Korea.

However, these results may be limited by the nature of the data obtained from the KHPS. We used a cross-sectional study design, which cannot elucidate the causal relationship between poor medication adherence and shift work. When considering medication adherence, a longitudinal or experimental intervention study would be better for inferring causality between medication adherence and the working system. Another limitation is that we measured medication adherence using self-reported questionnaires; however, the MMAS-4 has acceptable reliability and validity for measuring medication adherence^14^.

## Conclusion

The current study aimed to assess the association between medication adherence to chronic diseases and working schedules. Workers with a shift-work schedule are vulnerable to poor adherence to medication for chronic diseases. Appropriate medication adherence is a key factor in the management of chronic disease. Thus, the results of this study indicate that further studies regarding the role of work schedules are needed to prevent chronic diseases among workers.

### Ethics approval and consent to participate

This study was performed in accordance with the ethical standards of the Declaration of Helsinki (1964) and its subsequent amendments. The KHPS data were anonymized prior to their release to the authors. All the participants provided written informed consent. The institutional review board of the Gil Medical Center, Gachon University, approved this study (IRB number: GFIRB2022-045).

## Supplementary Information

Supplementary Information.

## Data Availability

The data are openly available in a public repository (Korea Health Panel Survey, https://www.khp.re.kr:444/eng/main.do).
