# Supplementary material for: Association between medication adherence to chronic diseases and shift-work schedules in the Korean working population
Source: Sci Rep. 2022 Dec 30;12:22595. doi: 10.1038/s41598-022-26618-9 (PMC9803658; doi:10.1038/s41598-022-26618-9)
Supplement: Supplementary file 1 — Supplementary Information. [file 41598_2022_26618_MOESM1_ESM.docx]

**Supplementary Tables**

| Supplementary Table 1. Results of a generalized estimating equation analyzing the risk of poor medication adherence | |
| --- | --- |
|  | Risk of poor medication adherence,  Odds Ratio (95% Confidence Interval) |
| Sex |  |
| Male | Reference |
| Female | **1.23 (1.12–1.35)** |
| Educational status |  |
| Middle school | Reference |
| High School | 0.98 (0.88–1.09) |
| College or higher | 1.11 (0.95–1.30) |
| Household income level |  |
| 1^st^ quintile | Reference |
| 2^nd^ quintile | 1.02 (0.89–1.17) |
| 3^rd^ quintile | 0.88 (0.76–1.01) |
| 4^th^ quintile | 0.89 (0.77–1.03) |
| 5^th^ quintile | 0.89 (0.77–1.04) |
| Occupational classification |  |
| Office | 1.21 (1.00–1.43) |
| Service and sales | 1.17 (1.00–1.35) |
| Agriculture, forestry, and fishing | Reference |
| Manual | **1.27 (1.13–1.43)** |
| Working schedule |  |
| Daytime-fixed | Reference |
| Shift | **1.16 (1.02–1.33)** |
| *Bold indicates statistical significance.  †All results are adjusted for age in observation year, sex, education, household income level, occupational classification, and working schedule. | |

| Supplementary Table 2. General characteristics of the study participants with chronic diseases according to medication adherence with gender stratification. | | | |
| --- | --- | --- | --- |
|  | Medication adherence, person-year (% of row) | | *P*-value |
|  | Good | Poor |  |
| Men | 3,032 (67.3) | 1,476 (32.7) |  |
| Age (years) |  |  | 0.0016 |
| ≤ 40 | 299 (61.1) | 190 (38.9) |  |
| 41–60 | 1,572 (67.1) | 771 (32.9) |  |
| > 60 | 1,1,61 (69.3) | 515 (30.7) |  |
| Educational status |  |  | 0.0677 |
| Middle school | 1,001 (68.5) | 460 (31.5) |  |
| High School | 1,083 (67.9) | 513 (32.1) |  |
| College or higher | 948 (65.3) | 503 (34.7) |  |
| Household income level |  |  | 0.0402 |
| 1^st^ quintile | 123 (61.5) | 77 (38.5) |  |
| 2^nd^ quintile | 548 (64.6) | 300 (35.4) |  |
| 3^rd^ quintile | 812 (68.0) | 382 (32.0) |  |
| 4^th^ quintile | 783 (68.9) | 353 (31.1) |  |
| 5^th^ quintile | 766 (67.8) | 364 (32.2) |  |
| Occupational classification |  |  | 0.1211 |
| Office | 878 (66.0) | 452 (34.0) |  |
| Service and sales | 163 (63.7) | 93 (36.3) |  |
| Agriculture, forestry, and fishing | 76 (68.5) | 35 (31.5) |  |
| Manual | 1,915 (68.1) | 896 (31.9) |  |
| Working schedule |  |  | 0.0272 |
| Daytime-fixed | 2,566 (67.9) | 1,211 (31.1) |  |
| Shift | 466 (63.7) | 265 (36.3) |  |
| Women | 4,510 (64.9) | 2,442 (35.1) |  |
| Age (years) |  |  | <0.0001 |
| ≤ 40 | 272 (54.7) | 225 (45.3) |  |
| 41–60 | 2,004 (64.1) | 1,124 (35.9) |  |
| > 60 | 2,234 (67.2) | 1,093 (32.8) |  |
| Educational status |  |  | <0.0001 |
| Middle school | 3,105 (66.3) | 1,577 (33.7) |  |
| High School | 1,009 (63.3) | 584 (36.7) |  |
| College or higher | 396 (58.5) | 281 (41.5) |  |
| Household income level |  |  | 0.0289 |
| 1^st^ quintile | 1,014 (66.8) | 503 (33.2) |  |
| 2^nd^ quintile | 1,202 (64.5) | 661 (35.5) |  |
| 3^rd^ quintile | 914 (66.3) | 464 (33.7) |  |
| 4^th^ quintile | 729 (63.0) | 428 (37.0) |  |
| 5^th^ quintile | 651 (62.8) | 386 (37.2) |  |
| Occupational classification |  |  | 0.7846 |
| Office | 508 (61.3) | 320 (38.7) |  |
| Service and sales | 994 (65.0) | 536 (35.0) |  |
| Agriculture, forestry, and fishing | 1,368 (69.8) | 593 (30.2) |  |
| Manual | 1,640 (62.3) | 993 (37.7) |  |
| Working schedule |  |  | 0.4680 |
| Daytime-fixed | 4,263 (65.0) | 2,298 (35.0) |  |
| Shift | 247 (63.2) | 144 (36.8) |  |
|  | | | |

| Supplementary Table 3. Results of a generalized estimating equation analyzing the risk of poor medication adherence according to working schedule with gender stratification. | |
| --- | --- |
|  | Odds ratio (95% confidence interval) |
| Men |  |
| Daytime-fixed | Reference |
| Shift | 1.27 (1.07-1.51) |
| Women |  |
| Daytime-fixed | Reference |
| Shift | 1.05 (0.85-1.30) |
| All results are adjusted for age in observation year, education, household income level, occupational classification, and working schedule. | |
